# Stationary Rossby waves dominate subduction of anthropogenic carbon in the Southern Ocean

**DOI:** 10.1038/s41598-017-17292-3

**Published:** 2017-12-06

**Authors:** C. E. Langlais, A. Lenton, R. Matear, D. Monselesan, B. Legresy, E. Cougnon, S. Rintoul

**Affiliations:** 1 CSIRO Oceans and Atmosphere, Castray Esplanade, Hobart, TAS 7000 Australia; 2 Antarctic Climate and Ecosystems Cooperative Research Centre, University of Tasmania Private Bag 80, Hobart, Tasmania 7001 Australia; 3 Centre for Southern Hemisphere Oceans Research, CSIRO Castray Esplanade, Hobart, TAS 7000 Australia; 4 Institute of Marine and Antarctic Studies, University of Tasmania, IMAS – Hobart Private Bag 129, Hobart, TAS 7001 Australia

## Abstract

The Southern Ocean has taken up more than 40% of the total anthropogenic carbon (C_ant_) stored in the oceans since the preindustrial era, mainly in subantarctic mode and intermediate waters (SAMW-AAIW). However, the physical mechanisms responsible for the transfer of C_ant_ into the ocean interior remain poorly understood. Here, we use high resolution (1/10°) ocean simulations to investigate these mechanisms at the SAMW-AAIW subduction hotspots. Mesoscale Stationary Rossby Waves (SRWs), generated where the Antarctic Circumpolar Current interacts with topography, make the dominant contribution to the C_ant_ transfer in SAMW-AAIW in the Indian and Pacific sectors (66% and 95% respectively). Eddy-resolving simulations reproduce the observed C_ant_ sequestration in these layers, while lower spatial resolution models, that do not reproduce SRWs, underestimate the inventory of C_ant_ in these layers by 40% and overestimate the storage in denser layers. A key implication is that climate model simulations, that lack sufficient resolution to represent sequestration by SRWs, are therefore likely to overestimate the residence time of C_ant_ in the ocean, with implications for simulated rates of climate change.

## Introduction

By sequestering more than 25% of anthropogenic CO_2_ emissions every year, the oceans mitigate the rate of climate change^1,2^. The Southern Ocean in particular is an important contributor to anthropogenic carbon (C_ant_) sequestration, accounting for approximately 40% of the total ocean uptake^1,3,4^. Despite the importance of the Southern Ocean, the physical mechanisms responsible for carbon exchanges between the well-ventilated surface mixed-layer and the ocean interior and the pathways by which carbon (natural and anthropogenic) is sequestered into the ocean interior remain poorly understood^2,5–7^. At present our understanding of carbon uptake and storage is based on sparse observations and coarse resolution models^6,8,9^. There is a need to evaluate how eddies may alter the uptake and storage.

In the Southern Ocean density surfaces shoal southwards across the ACC, which exposes dense waters to the atmosphere and connects the surface layer with the ocean interior. Heat, oxygen and C_ant_ are efficiently taken up by the ocean near the Polar front because the upwelled waters are constantly transported away from the uptake zone through Ekman transport^10^. Further north, waters sinking on the northern flank of the ACC transfer oxygen, heat and C_ant_ into the ocean interior along isopycnal layers that deepen as the water moves north. This transfer, called subduction, is the rate-limiting step for the sequestration of C_ant_ in the deep ocean^8^. Subduction of Subantarctic mode and Antarctic intermediate waters (SAMW-AAIW) constitutes the upper limb of the global overturning circulation and makes the largest contribution to the uptake and storage of C_ant_ by the Southern Ocean^9,11,12^. Subduction does not happen uniformly along the circumpolar outcrop of density surfaces. Bathymetrically constrained hotspots control the pathways connecting the well-ventilated mixed-layer and the ocean interior^8,13^. Understanding the mechanisms behind the localized subduction is of great importance as the ability of the ocean to sequester and store C_ant_ is set by this exchange^8,14–17^. Sallée *et al*.^8^ showed that the interplay of the horizontal transport with variations in winter mixed-layer depth (i.e. lateral induction) produced localized subduction hotspots. However, this conclusion was based on coarsely gridded time-averaged observations of the ocean dynamics. As eddy processes are hypothesised to be important for subduction^18^, there is clearly a need to investigate the contribution of mesoscale circulation to C_ant_ subduction in mode and intermediate waters.

Efforts in recent years have investigated the direct impact of mesoscale eddies on biogeochemical cycles and carbon fluxes, showing that eddy-driven transport tends to compensate the wind-driven vertical transport^8,19,20^, and quantifying the eddy contribution to advection and mixing of tracers^15,17,20–22^. However, an important characteristic of eddy-resolving models, satellite and *in-situ* observations that is yet to be explored is the presence of standing meanders downstream of topographic obstacles (Supplementary Information Fig. 1)^20,23–30^. These meanders, which are poorly represented in coarse resolution models (lower than 1/2° resolution), can significantly affect the vertical and horizontal transport, with vertical transports up to ten times stronger than Ekman pumping and northward deviations from zonal flow of up to 50° angle in eddy-resolving models and observations (Supplementary Information Fig. 1)^29,31^. Hughes^23,24^ showed that these stationary meanders are consistent with the dynamics of stationary equivalent-barotropic Rossby waves (SRWs).

With increasing computational power, it is now possible to run high resolution (1/10°) eddy resolving biogeochemical ocean simulations^20,32,33^ and quantify the contribution of mesoscale processes to C_ant_ subduction (see Methods). Here, we show that localized subduction of C_ant_ occurs where the SRWs interact with the sloping base of the winter mixed-layer in SAMW-AAIW density classes. The simulation, with sufficient spatial resolution to realistically capture SRWs, transports more C_ant_ in the intermediate density layers than a coarse resolution model of the type used in CMIP5, which poorly resolves SRWs and sequesters C_ant_ in denser layers.

## Results

We investigate the surface to interior pathways of C_ant_ in the Southern Ocean using 1/10° resolution biogeochemical ocean simulations^32,33^ (see Methods). At this resolution, mesoscale eddies are fully resolved, while 1/4° resolution simulations are only eddy permitting and 1/2° and lower resolution simulations do not explicitly resolve eddies and need parameterizations of their effect on the circulation. The model circulation, the meandering of fronts and associated vertical velocities, the winter mixed-layer depth and the C_ant_ distribution and storage in the Southern Ocean are all in good agreement with observations (see Supplementary Notes 1). While the simulation has a deeper winter mixed-layer depth than calculated from the Argo-only CARS climatology (updated to 2015)^34,35^, importantly the spatial patterns are well represented with deep mixed-layers found north of the Subantarctic Front in the south-east Indian-Ocean and in the central-east Pacific (Supplementary Information Fig. 2). The simulation also realistically reproduces the observed spatial distribution of C_ant_ from GLODAP^36^, with C_ant_ entering the ocean interior along particular isopycnals in each basin (Supplementary Information Fig. 3). The good agreement with the observations supports the use of the high-resolution simulation to investigate the physical processes responsible for localized C_ant_ subduction in SAMW-AAIW.

**Figure 1:**
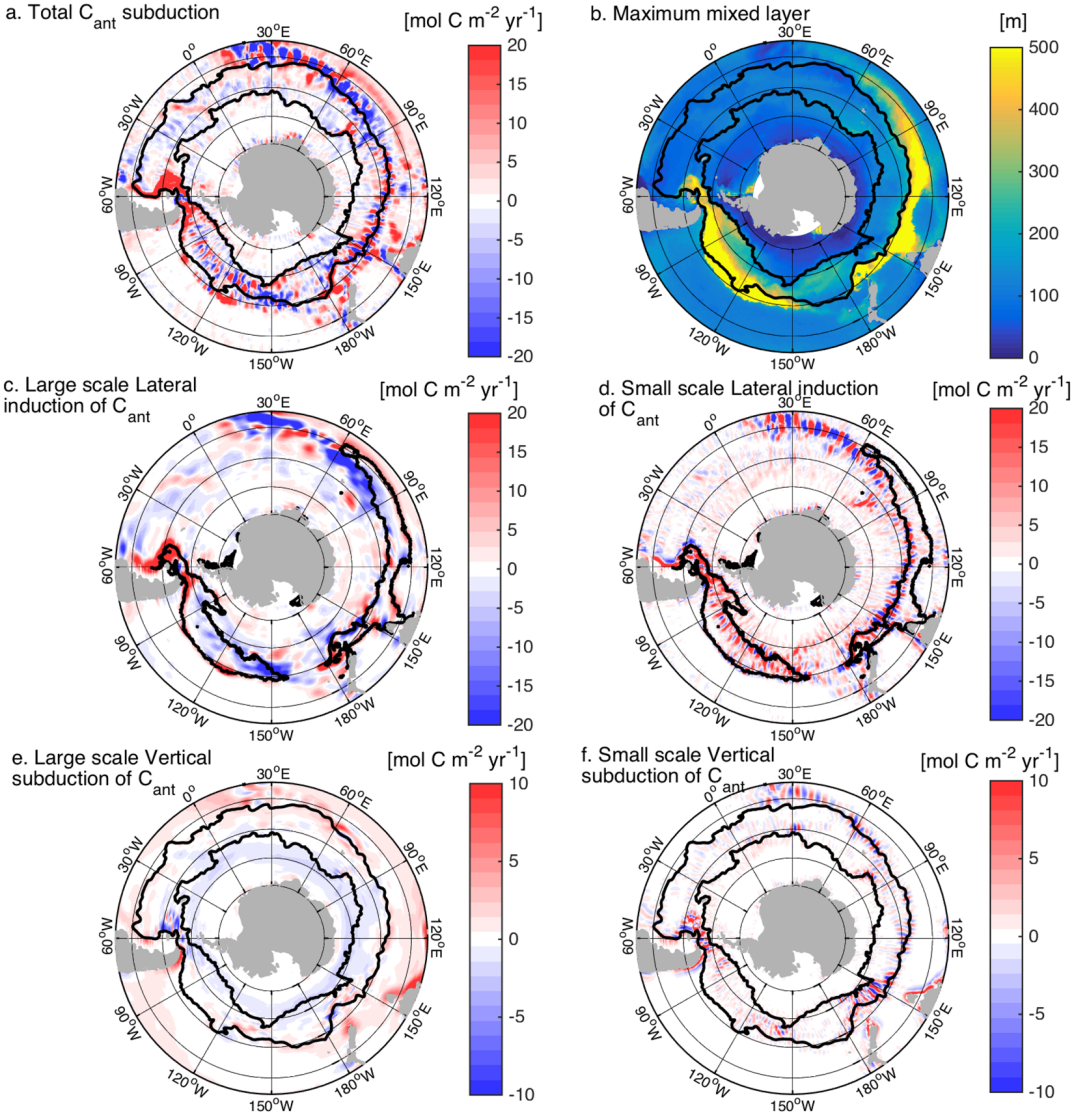
Simulated C_ant_ subduction into (+) and out (−) of the ocean interior at the base of the winter mixed-layer for (**a**) total subduction, (**c**) and (**d**) lateral induction, (**e**) and (**f**) vertical subduction (Ekman pumping and vertical eddy subduction). The simulated maximum climatological winter mixed-layer depth is shown in (**b**). The lateral induction and vertical subduction are separated into large- and small-scale contributions, using a Gaussian smoothing window with a decorrelation radius of 200 km. Black lines in (**a**), (**b**), (**e**) and (**f**) show the mean position of the Subantarctic and Polar Fronts. Black contours in (**c**) and (**d**) delineate the position of the 300 m maximum winter mixed-layer depth. Figures are plotted using MATLAB R2015a (http://www.mathworks.com/). The maps are generated using M_Map (a mapping package, http://www.eos.ubc.ca/~rich/map.html).

**Figure 2:**
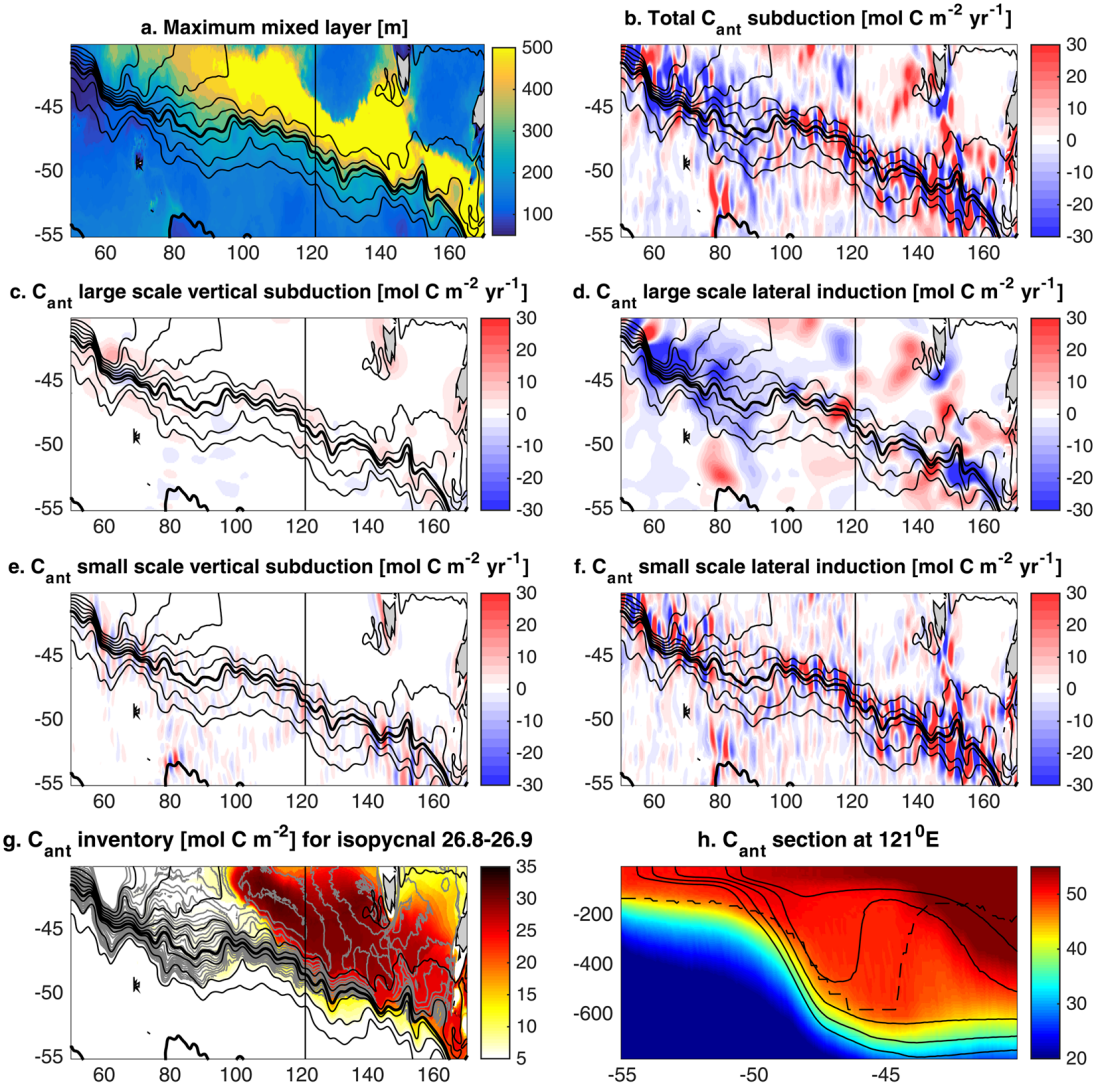
Stationary Rossby waves and C_ant_ subduction in the Southeast Indian ocean. (**a**) Winter maximum mixed-layer. C_ant_ transfers into (+) and out of (−) the ocean interior at the base of the winter mixed-layer depth for (**b**) total C_ant_ subduction, (**c**) and (**e**) vertical subduction and (**d**) and (**f**) lateral induction. The lateral induction and vertical subduction are separated into large- and small-scale contributions, using a smoothing window with a decorrelation radius of 200 km. (**g**) Inventory of C_ant_ in the ocean interior along isopycnals 26.8 to 26.9 kg m^−3^. Black contours in (**a**) to (**g**) show SSH contours approximating the ACC with the SAF and PF in bold. Vertical black line in (**a**) to (**g**) shows the position of the section represented in (**h**). Grey contours in (**g**) show the approximate geostrophic stream-function along isopycnals 26.8–26.9 kg m^−3^ as defined by^58^. (**h**) shows C_ant_ concentration in mmol.m^−3^ along 121°E with isopycnal contours from 26.8 to 27 kg m^−3^ every 0.05 (black contours) and maximum mixed-layer depth (dashed line). Figures are plotted using MATLAB R2015a (http://www.mathworks.com/).

**Figure 3:**
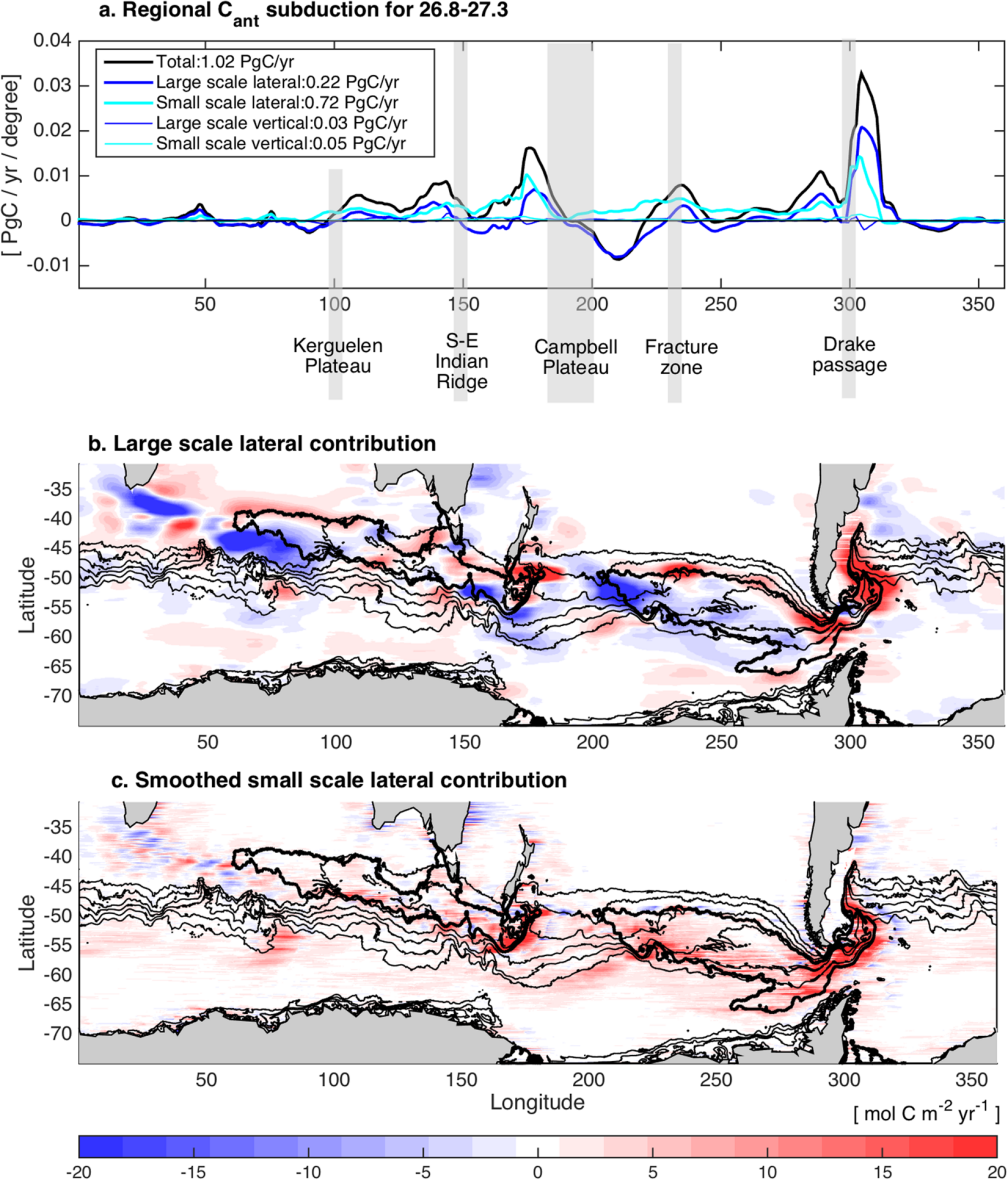
Localized C_ant_ subduction induced by Stationary Rossby Waves: (**a**) separation of the subduction along the circumpolar belt in the density range of SAMW-AAIW 26.8–27.3 into different contributions, (**b**) large sale lateral contribution to C_ant_ subduction as in Fig. 1c, (**c**) smoothed small scale lateral contribution to C_ant_ subduction as in Fig. 1d. Black contours in (**b**) and (**c**) delineate the position of the 300 m maximum winter mixed-layer depth (bold contours), and the density contours from 28.7 to 28.3 kg.m^−3^ (thin contours) which is the density range used in (**a**). Major topographic obstacles are identified in (**a**). Figures are plotted using MATLAB R2015a (http://www.mathworks.com/).

C_ant_ subduction south of 30°S is calculated as the transfer of C_ant_ through the bottom of the climatological winter mixed-layer, which captures the subduction into the ocean’s permanent thermocline (Fig. 1a and b) (see Methods). To better understand the transfer mechanisms, the physical subduction is separated into large-scale and small-scale contributions to horizontal and vertical transfers (Fig. 1c to f), the separation between large and small scales being 200 km (see Methods and Supplementary Methods 1 for the choice of separation scale). Each term is then multiplied by C_ant_ and normalized, so that the sum of the contributions equals the total C_ant_ subduction. The subduction is dominated by the horizontal contributions. Spatial patterns of the large-scale contribution to C_ant_ subduction are in agreement with results based on sparse observations^8^. The large-scale horizontal transfer occurs at the eastern and western ends of the deep mixed-layer pool where the current interacts with a deepening mixed-layer base (at around 70°E and 150°W) or a shoaling mixed-layer base (at around 180°W and 60°W) (Figs 1c and 2d, Supplementary Information Figs 6a and 7b). At the large scale, there is also some vertical subduction resulting from a compensation between Ekman pumping and large-scale eddy effects^8^, with some localized bottom torque effects where the ACC interacts with topography^25^ (Figs 1e and 2c, Supplementary Information Figs 6g and 7f). For the 1981–2012 period, the large-scale contributions result in a net subduction of 0.24 PgCyr^−1^ in SAMW-AAIW density classes (26.8–27.3 kg m^−3^) south of 30°S, with the horizontal contribution dominating (Fig. 3a). This agrees with estimates using sparse observations (0.23 ± −0.15 PgCyr^−1^ in^8^) and a coarse resolution model (0.19 PgCyr^−1^ in^37^). However, in our 1/10° simulation, the large-scale subduction contributes only 24% of the total net subduction of C_ant_ to the ocean interior for the 1981–2012 period. In SAMW-AAIW density classes the total net subduction reaches 1.02 PgCyr^−1^, with 76% (0.78 PgCyr^−1^) induced by the small scale circulation (Fig. 1d and f), while the large scale transfer accounts for the remaining 24% (0.24 PgCyr^−1^) (Fig. 3a). While our estimate is larger than the Sallée *et al*. estimate for 1995^8^, the modelled 0.82 PgCyr^−1^ air-sea C_ant_ uptake between 70–40°S (1.04 PgCyr^−1^ south of 30°S, our area of interest) is in the range of the current best estimates of 0.72 to 1.04 (0.92 ± 0.24) PgCyr^−1^ ^1^. As in^10^, this uptake is mainly happening near the divergence zone where upwelled water can take up a large amount of C_ant_ (not shown). Our results imply that subduction estimates based on limited observations as in^8^ or non-eddy resolving simulations underestimate the C_ant_ subduction rate by SAMW-AAIW.

Why does increased resolution lead to greater C_ant_ uptake in SAMW-AAIW? When we compare the time mean spatial patterns of small scale subduction of C_ant_ with those derived from observations^8^ we see large differences (Fig. 1d and f and Fig. 1 in^8^). Of particular interest, three locations show wavelike patterns of C_ant_ transfers in and out of the mixed-layer with wavelengths of 300 to 500 km and C_ant_ subduction of more than ± 20 molCm^−2^yr^−1^, downstream of the Kerguelen Plateau (100–140°E), the South-east Indian Ridge (150–180°E), and the Pacific Fracture Zone (90–140°W) (Figs 1 and 2, Supplementary Information Fig. 6 for the physical subduction terms only and Fig. 3a for the position of the topographic obstacles). These intense transfers occur where the SAMW-AAIW density classes outcrop at the base of the winter mixed layer, with lighter density classes in the Indian Ocean and denser ones in the Pacific (Supplementary Information Fig. 8). Spatially averaging these small scale C_ant_ transfers reveals that their overall effect is a transfer of C_ant_ from the mixed layer to the interior where isopycnals outcrop along the southern edge of the deep mixed layer (Fig. 3c). The large scale contribution shows hot spots of subduction and reventilation along the circumpolar path, with subduction mainly happening at the northern and eastern edge of the deep mixed layer. At the subduction hotspots and for the 27.8–27.3 (kg/m^3^) density range, small scale lateral subduction contributes from 40% of the total localized C_ant_ transfer at Drake passage to 66% between 90–150°E in the Indian sector and 95% between 220–280°E in the Pacific (Fig. 3). The along-isopycnal geostrophic flow then carries C_ant_ from the localised subduction sites into the interior (Fig. 2g and Supplementary Information Fig. 8).

The wavelike transfers are coincident with the ACC standing meanders. Downstream of topographic obstacles, the main jets of the ACC act as waveguides. The wavelengths of the standing meanders agree with the linear approximation of a simple one layer quasi-geostrophic (barotropic) model of SRWs (e.g.^38^), where ${L}_{S}=2\pi /\sqrt{\beta /\bar{u}}$, with $\beta =2\Omega \,\cos (\theta )/a$, Ω the angular speed of the Earth’s rotation, *a* the mean radius of the Earth and *θ* the latitude, and $\overline{u}$ is taken as the barotropic component of the jet velocity (the average velocity between 1000 m and the bottom) (see Supplementary Methods 2 for a discussion of the theory and typical range of $\overline{u}$). In satellite observations and 1/10° resolution model outputs, the SRWs are a prominent feature of the mean ACC^24^ (Supplementary Information Fig. 1d). Observations show that these meanders can induce strong vertical velocities and “frontal subduction”^29,30,39^.

**Figure 4:**
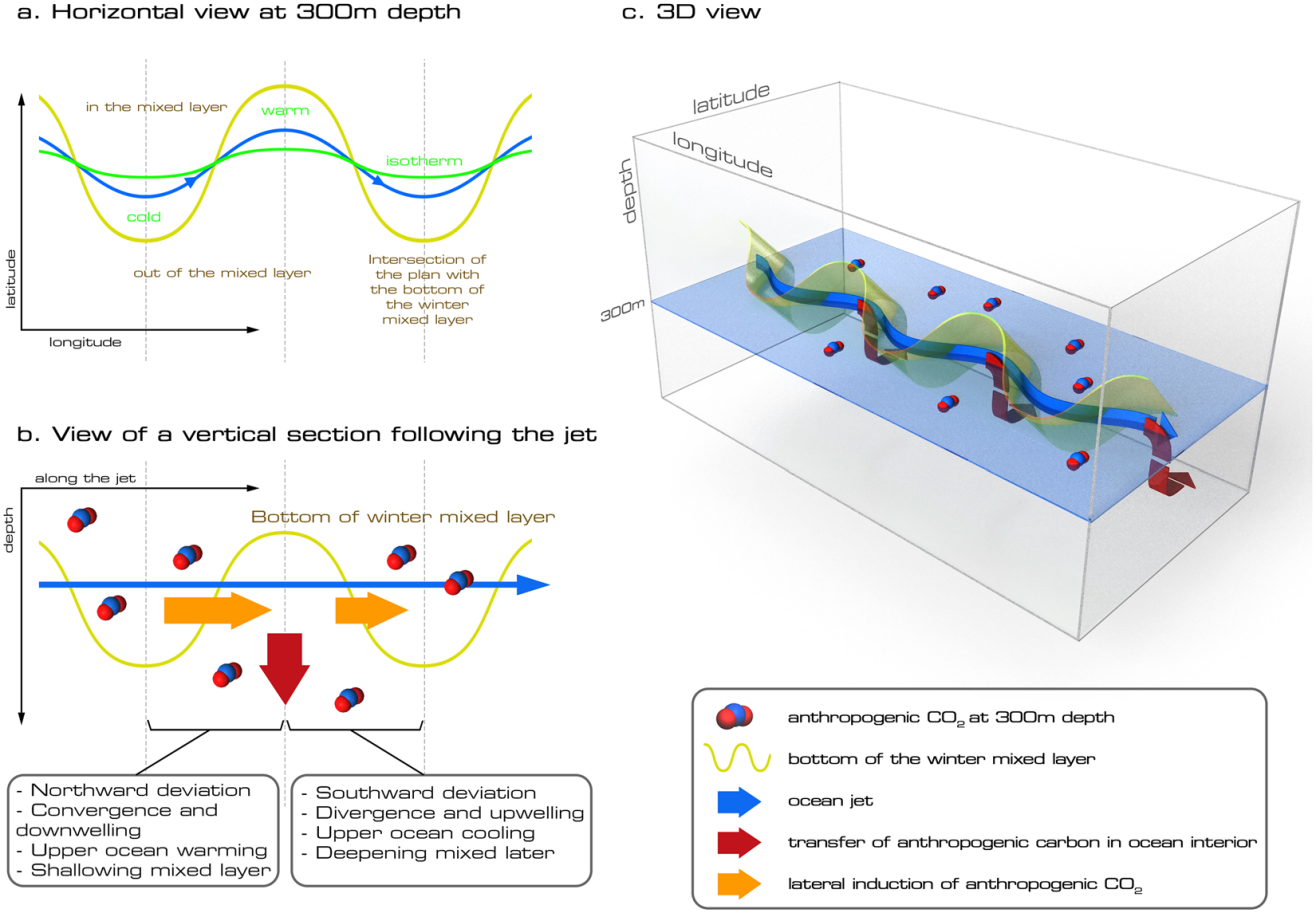
Schematic of the Stationary Rossby Wave-induced anthropogenic carbon transfer: 3 different views of the transfer: horizontal plan (**a**), vertical section along the meandering jet (**b**) and 3D view (**c**). The meander and the base of the mixed-layer are not aligned (**a**), with a shallowing and deepening of the mixed layer along the jet (**b**). The change in mixed layer along the jet is due to warm (cold) water intrusion near the surface during the northward (southward) deviation of the meander. These intrusions result in a misalignment between the jet and surface isotherms (**a**). The interaction of the meander with the base of the winter mixed layer transfers fluid in and out of the ocean interior (blue arrows). C_ant_–rich waters are injected below the mixed layer during the downwelling/northward deviation of the meander, and C_ant_-low water are pushed out of it during the upwelling/southward deviation (pink arrows in (**b**), which result in a net flux of C_ant_ into the interior (red arrows in **b** and **c**). Figures are plotted using Adobe photoshop (http://www.adobe.com/au/products/photoshop.html?promoid=V6NZKW75&mv=otherl).

SRWs transfer C_ant_ in and out of the mixed-layer by both vertical subduction and lateral induction (Figs 1e and f, 2e and f, Supplementary Information Fig. 6c and i). As the southward and northward deviations of the jets of the ACC interact with the sloping mixed-layer base, water is pushed in and out of the mixed-layer along the southern edge of the deep mixed-layer pool in the Indian sector (100–180°E), and in the Pacific sector (90–140°W). These transfers occur mainly downstream of the points of interaction of the ACC jets with topography where the meanders freely develop, with the exception of the Campbell Plateau where steering occurs (Fig. 3). The transfers are due to a misalignment between the ACC jets and the base of the mixed-layer (Fig. 4). While the base of the mixed layer meanders with the same phase as the ACC, its meandering amplitude is larger than the ACC leading to transfers out of the mixed layer during northward deviations and transfers into the mixed layer during southward deviations (Fig. 4). In other words, along the meandering jet, there is a shallowing of the base of the mixed layer during the northward deviation and a deepening during the southward deviation. As the surface mixed-layer has a higher concentration of C_ant_ than the ocean interior (Fig. 2f), the interaction of SRWs with the sloping mixed-layer base results in a net C_ant_ transport into the ocean interior between 100–180°E and between 90–140°W (Fig. 3a and c, red arrows in Fig. 4). During the downwelling/northward deviation, C_ant_–rich waters are injected in the SAMW-AAIW density layer below the mixed layer, while during the upwelling/southward deviation, older C_ant_–low waters are reventilated. The SRWs are at the same time associated with upwelling and downwelling along the ACC path (Figs 1f and 2e, Supplementary Information Fig. 6i). While there is a net C_ant_ subduction associated with the vertical advection, it is an order of magnitude smaller than the C_ant_ subduction associated with lateral induction (Fig. 3). Consistent with previous studies^39–41^, we find that transient eddies only contribute approximately 13% of C_ant_ subducted in intermediate and mode waters (26.8–27.3 kg m^−3^) (see Supplementary Notes 2).

## Discussion

**Figure 5:**
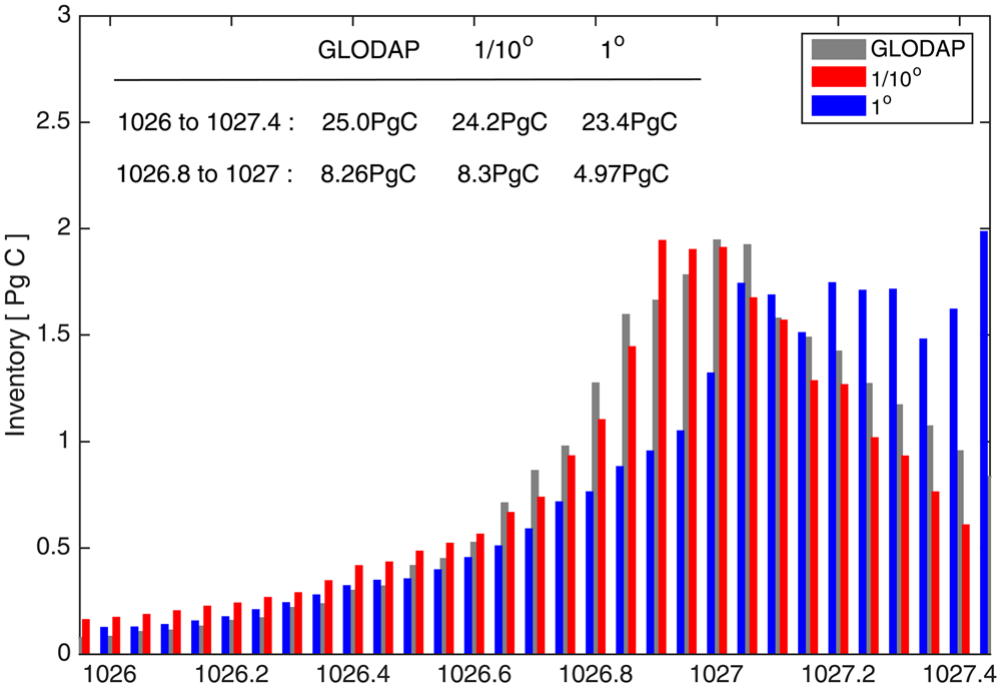
C_ant_ inventory distribution per density classes: inventory between 30°S and the PF for comparison between GLODAP^36^ (grey bars), 1/10° model (red bars) and 1° model (blue bars) (see Supplementary Notes 1 for more details about inventories per basins). Due to how the biogeochemical models were initialised (1992, see Methods), the 1995 observations are compared with the 2014 C_ant_ inventory in the models, focussing on the inventory away from the surface (below 100 m). While the total amount of C_ant_ in the Southern Ocean is quite similar in all models and observations, the low resolution model underestimates by 40% the C_ant_ sequestered in the 26.8 to 27 density layers, due to sequestration in denser density classes.). Figures are plotted using MATLAB R2015a (http://www.mathworks.com/).

SAMW-AAIW (26.8–27.3 density range) sequester the largest quantities of C_ant_ in the Southern Ocean^12^, accounting for 56% of the carbon inventory south of 30°S in the high-resolution model and 55% in the observations (Fig. 5). In the high-resolution model, SRWs make the dominant contribution to subduction of C_ant_ in the density range of SAMW-AAIW. With wavelengths ranging from 300 to 500 km, the standing meanders are present in 1° resolution models, but with small amplitude (Supplementary Information Fig. 1e). Coarse resolution ocean simulations have only weak SRWs (Fig. 2a and b in^42^), resulting in little contribution to C_ant_ subduction (Supplementary Information Fig. 7). Higher resolution captures the important gradients that define fronts, jets and sharp horizontal and vertical structures (Supplementary Information Fig. 1). Hence, barotropic and baroclinic instabilities are better modelled and the destabilization of the sharp jets generates eddies, allowing for more eddy-mean flow non-linear interactions. As a consequence, the core of the standing meanders can be accelerated by convergence of eddy kinetic energy^28^. In this way, mesoscale eddies maintain the standing meanders downstream of topographic obstacles, which may affect the amplitude of the waves and then indirectly contribute to C_ant_ subduction. While the impact of transient eddies on large scale circulation can be parameterized in low resolution simulations^43,44^, their effect on frontogenesis is not. Therefore, eddy-resolving resolution is needed to represent sharp jets, SRWs and the potential eddy-mean flow interaction.

In both high- and low-resolution simulations and observation-based estimates, advection is the dominant mechanism for the transfer of C_ant_ through the base of the winter mixed-layer, with the lateral induction term dominating over vertical subduction. However, changes in resolution modify where and how advection operates. In the high-resolution model, SRWs subduct C_ant_ on the southern sides of the deep mixed-layer pools where SRWs interact with the sloping mixed-layer base. In low-resolution models and estimates based on sparse observations, the C_ant_ subduction occurs predominantly at the eastern and western end of each basin, and on the northern side of the deep mixed-layer. Transport mechanisms do not operate in the same manner in high- and low-resolution ocean.

It is the misalignment between the meandering ACC jets and the base of the mixed-layer that is central to the SRWs-induced transfer described here (Fig. 4). Specifically, horizontal transfers in and out of the mixed layer occur because of a shallowing and deepening of the mixed layer along the jet (Fig. 4b). We hypothesise that the change of mixed layer depth along the jet is due to cross-frontal transfer induced by the rotation of the horizontal flow with depth along the meander as previously described^29,45^. The departure from equivalent barotropic behaviour in the meander of the ACC jet causes the horizontal velocity to rotate to the right in the upper ocean and then to the left in the intermediate water during the northward deviation, in a process called backing. In the upper ocean the slight rotation to the right causes the transport of “warm” surface water across the ACC jet. The opposite is true during the southward deviation and is called veering (Fig. 4a). This warm or cold water transport across the ACC jet in the upper ocean modifies the upper ocean stratification and the mixed layer depth along the jet. As a consequence, the base of the mixed layer, that delimits C_ant_–rich waters, is not aligned with the meandering jet, and C_ant_–rich waters are injected in the SAMW-AAIW density layer below the mixed layer. Once in the interior, C_ant_–rich waters are mixed along their density layer. Backing and veering can have a substantial influence on cross-frontal poleward heat fluxes when integrated over the water column^29,45^. Here we show that they can also influence the alignment of the based of the mixed layer with the meandering ACC jets, influencing flux into the ocean interior.

As SRWs are only adequately resolved in the high resolution simulation (Supplementary Information Fig. 1,^42^), the transfer mechanisms act on slightly different density classes in the high- and low-resolution models and the amount of C_ant_ injected into the different density classes changes with model resolution. The high-resolution model transports almost twice as much C_ant_ into the ocean interior in the 26.8–27.3 density range (Supplementary information Table 1). To assess the ocean interior distributions, we compare the inventory of C_ant_ in density classes with the observed values^36^ (Fig. 5). Due to how the high-resolution biogeochemical model was initialised (1992, see Methods), the 1995 GLODAPv1 C_ant_ inventory is compared with the 2014 C_ant_ inventory in the models, focussing on the inventory away from the surface (below 100 m). Both the observations and high-resolution simulation have a well-defined peak in C_ant_ storage at a density of 26.8 kg m^3^ (Fig. 5). While the total amount of C_ant_ in the Southern Ocean and the partitioning per latitude are quite similar between models and the observations (as in^46^) (Supplementary Information Fig. 9), the density distribution is better represented by the high-resolution model than the low resolution model (Fig. 5). The storage maximum occurs at higher density in the low-resolution simulation, and C_ant_ is distributed across a broader range of densities (26.8 to 27.5 kg m^3^) (Fig. 5). The density shift is directly linked with the subduction processes in the low resolution model which act on denser density classes and occur in different locations than in the high resolution model. As a result, we find an underestimation of C_ant_ inventory ($\approx$ 40%) in the 26.8 to 27 kg.m^−3^ density range in the low resolution model compared to observations. This result is consistent with too little subduction in this density range in low resolution climate models used in the Intergovernmental Panel on Climate Change 4^th^ and 5^th^ Assessment Reports^47,48^. The underestimate of water mass formation has previously been linked to the effect of transient eddies in the Northern Hemisphere^49^, or to a misrepresentation of the mixed layer^50^. Here we show that SRWs, largely absent in low resolution climate models, are required to reproduce the observed distribution of C_ant_ as a function of density in the Southern Ocean.

These results also have implications for C_ant_ pathways and residence times. As the low resolution model subducts C_ant_ on denser isopycnals, the C_ant_ inventory is deeper in the water column and south of where it is in the high resolution simulation, especially in the Pacific (Supplementary Information Figs 4 and 5). This denser and deeper pool of C_ant_ in the coarse resolution simulation is then more isolated from the C_ant_-rich subtropical mode waters, which are lighter than 26.5 in all the models experiments and observations (Supplementary Information Fig. 5). Transfers between SAMW and subtropical waters are thought to be critical for the global carbon cycle^9^. A lighter ventilated layer in the high resolution model would probably result in more connection with the subtropical waters. Once injected into the ocean interior, the C_ant_ distribution is controlled by along-isopycnal flow in large scale gyres that cover hundreds of kilometres (grey contours in Fig. 2g). Different water masses will ventilate the deep ocean at different time scales from decadal to centennial and resurface in different regions^51,52^.

The Southern Ocean overturning circulation is the critical large-scale oceanographic feature sustaining the uptake of anthropogenic carbon^8–10^. In particular, the rate-limiting step in the transfer of C_ant_ from the atmosphere to the deep ocean has been shown to be the transfer at the bottom of the mixed-layer^8^. Here we show that knowledge of the physical mechanisms responsible for C_ant_ transfer between the well-ventilated surface mixed-layer and the ocean interior is key to understanding the density distribution of past and future anthropogenic carbon uptake by the ocean. Further, the localized nature of the C_ant_ sequestration suggests that an observational effort, focused on the regions where SRWs occur, is needed to better constrain C_ant_ sequestration. An important remaining question will be understanding how future changes in the strength and position of the ACC and changes in mixed layer depth will impact SRWs and the subduction of C_ant_ in SAMW-AAIW.

## Methods

### Ocean and BGC models

To illustrate the importance of model resolution on C_ant_ subduction, 1° and 1/10° biogeochemical ocean simulations are compared. 1/10° simulations fully resolve mesoscale eddies, while 1° simulations do not explicitly resolve eddies and need parameterizations. Both simulations are based on version 4p1d of the Geophysical fluid Dynamics Laboratory Modular Ocean Model^53^, and include the World Ocean Model of Biology And Trophic dynamics (WOMBAT)^32,54^. WOMBAT is based on a simple nutrient, phytoplankton, zooplankton and detritus model, with the addition of an oxygen and carbon cycle. C_ant_ is calculated as the difference between two tracers of Dissolved Inorganic Carbon, one that sees an (pre-industrial) atmospheric value of 280 ppm (natural carbon tracer), and a second tracer that sees the observed rising atmospheric CO_2_ concentration (total carbon tracer).

The 1° and 1/10° simulations span the period 1979 to 2014, forced by 3-hourly Japanese 55-year Reanalysis (JRA-55^55^). A 20-year repeat of year 1979 has been used to spin-up the physical and biological fields. As the inclusion of the BGC component is computationally expensive, the BGC fields are only integrated between 1992–2014.

The 1° ocean simulation is based on the ACCESS-O configuration^56^, whereas the 1/10° simulation is based on the near-global Ocean Forecasting Australia Model configuration (OFAM3)^32^. In the Southern Ocean, the 1/10° OFAM3 configuration gives a resolution of 4.7 km at 65°S, 7.8 km at 45°S and a constant meridional resolution of 11 km. It has 51 vertical layers, with 14 layers between the surface and 100 m depth, and partial cells to better represent bottom topography. The 1/10° configuration has a good representation of the frontal structure and filamented nature of the ACC and captures much of the mesoscale variability and development of baroclinic eddies^27^. For more details about parameterizations and spin-up, please refer to Supplementary Methods 3.

### Subduction = Transfer through the base of the winter mixed-layer

We define the mixed-layer depth with a density difference from the surface of 0.03 kg m^−3^.

As we want to capture the subduction into the permanent thermocline, we use the maximum mixed-layer depth (MLD) from the model monthly climatology to calculate the transfer between the surface and the ocean interior following^8,57^ (see Supplementary Methods 1 for discussion of other methods).

As the winter mixed-layer deepens and shallows along the circumpolar path (Fig. 1b), the transfer through the base of the winter mixed layer has not only a vertical component but also an horizontal one. Following^8,57^, the mean subduction S is partitioned into a horizontal component ($\overline{U.}\nabla H$) due to the time-mean horizontal transport through the sloping mixed-layer base, the so-called lateral induction, and a vertical component ($\overline{w}$), vertical mixing being neglected, $\overline{.}$ being the time-average operator:

1 $$S=\overline{w}+\overline{U}\cdot \nabla H$$

The mean physical subduction is then separated into large and small scales contributions (subscripted L and S in equation 2) using a spatially isotropic low-pass Gaussian filter with a decorrelation radius of 200 km and a half-window size of 400 km which reduces the Gibbs effect (see Supplementary Methods 1 for more details on the size of smoothing windows and its relationship with physical flow).

The C_ant_ subduction S_Cant_ is therefore composed of four contributions:

2 $${S}_{Cant}=\overline{{w}_{L}}\cdot \overline{{C}_{ant}}+{(\overline{U}\cdot \nabla H)}_{L}\cdot \overline{{C}_{ant}}+\overline{{w}_{S}}\cdot \overline{{C}_{ant}}+{(\overline{U}\cdot \nabla H)}_{S}\cdot \overline{{C}_{ant}}$$

Equation (2) accounts for the sign of subduction: transport into the ocean interior uses the C_ant_ concentration in the mixed-layer and transport into the mixed-layer uses the C_ant_ concentration below the mixed-layer. The vertical components can be further broken down into Ekman pumping and eddy-induced vertical subduction (see Supplementary Methods 1 and Supplementary Information Figs 6 to 9).

The subduction is effectively a diapycnal flux as in^9^, with C_ant_ being transferred towards denser density classes through the base of the mixed layer.

### Data availability

This work used simulations performed within the frame of the “Ocean downscaling strategic project” funded by the CSIRO Oceans and Atmosphere Business Unit. Mean Dynamic Topography and geostrophic velocities were generated by DUACS and distributed by AVISO (ftp://ftp.aviso.oceanobs.com). GLODAP (GLobal Ocean Data Analysis Project) data were generated by the Carbon Dioxide Information Analysis Centre (http://cdiac.ornl.gov/oceans/glodap/GlopDV.html). CARS climatology (http://www.marine.csiro.au/~dunn/cars2009/) is a product of CSIRO Oceans and Atmosphere and was updated by DM using Argo only 2006–2015.

## Electronic supplementary material

Supplementary Information
