# Supplementary material for: Stationary Rossby waves dominate subduction of anthropogenic carbon in the Southern Ocean
Source: Sci Rep. 2017 Dec 6;7:17076. doi: 10.1038/s41598-017-17292-3 (PMC5719014; doi:10.1038/s41598-017-17292-3)
Supplement: Supplementary file 1 — Supplementary Information [file 41598_2017_17292_MOESM1_ESM.pdf]

Stationary Rossby waves dominate subduction of anthropogenic carbon in the Southern Ocean

C. E. Langlais [1*], A. Lenton [1,2,3], R. Matear [1], D. Monselesan[1], B. Legresy[1,2], E. Cougnon[1,4], S. Rintoul[1,2,3]

1. CSIRO Oceans and Atmosphere, Castray Esplanade Hobart TAS 7000 Australia

2. Antarctic Climate and Ecosystems Cooperative Research Centre, University of Tasmania Private Bag 80 Hobart Tasmania 7001 Australia

3. Centre for Southern Hemisphere Oceans Research, CSIRO Castray Esplanade Hobart TAS 7000 Australia

4. Institute of Marine and Antarctic Studies, University of Tasmania, IMAS – Hobart Private Bag 129 Hobart TAS 7001 Australia

* corresponding author clothilde.langlais@gmail.com CSIRO O&A Castray Esplanade Hobart 7000 TAS Australia

Supplementary Methods 1: **Physical transfers at the base of the winter mixed layer and scale separation**

Subduction rate can be calculated in many different ways, using lagrangian definition [59], using eulerian definition with variable mixed layer [16] or using an eulerian definition with a fixed mixed layer [8,57]. To capture the subduction into the permanent thermocline, we calculate the transfer through the fixed maximum mixed-layer depth following [8,57].

The annual mean physical subduction can be separated into three main mechanisms: vertical Ekman pumping, eddy-induced vertical subduction and lateral induction (Supplementary Information Fig. 8 and 9):

$$S = \overline{w_{Ek}} + \overline{w_{Eddy}} + \overline{U_H} \cdot \nabla H$$

In Sallée et al. [8,60], the eddy-induced vertical term is calculated using the GM parameterization and isopycnal slope. In our calculation, it is calculated as a residual (difference between total vertical and Ekman pumping).

Each physical subduction contribution can be separated into large and small scales contributions using a spatially isotropic low-pass filter (Supplementary Information Fig. 6 and 7). We take the weighting function as Gaussian in $\exp\left(d^2/_{rc^2}\right)$where d is the distance to the window centre and rc the decorrelation radius. To test the effect of the Gaussian smoothing window, we use two different decorrelation radius, 400 and 200km. In each case, the half window size is twice the size of the decorrelation radius to avoid Gibbs effect (800 and 400km respectively). The two smoothing are used in succession. The smoothed output of the 400km decorrelation radius smoothing is called the large scale. The residual is then smoothed using a 300km decorrelation radius. The smoothed output is called the medium scale and the residual the small scale.

This scale decomposition allows to view the mean ACC flow as being the association of the SRW and a flow with divergence associated with topographic features (as in [24]). In the 1/10° model, there is a clear scale separation between these two components. The spatial scale of the SRW is mainly smaller than 200km, while the spatial scale of the flow is larger than 400km (Supplementary Information Fig. 6). In-between 200 and 400km, the SRW imprint slightly on the lateral induction term. In the 1° model, the weak SRW has only on expression at medium scale, in-between 200 and 400km (Supplementary Information Fig. 7).

In light of these results and to separate the SRW from the large scale flow, we used a single Gaussian smoothing windows with a decorrelation radius of 200km (approximately 2°) in the main

text. The contribution of each spatial scales to the net C_{ant} subduction rate is shown in Supplementary Information table 1.

Supplementary Methods 2: **Standing Rossby Waves (SRWs)**

Circulation and SRWs in the models

In satellite observations and 1/10° resolution model outputs, the mean ACC flow can be viewed as the association of SRWs and a flow with divergence associated with topographic features [24]. SRWs with a wavelength of 200 to 500 km appear downstream of topographic obstacles, after steering accelerates the ACC jets: downstream of the Agulhas Plateau (30-60°E), downstream of the Kerguelen Plateau (100-140°E), downstream of the crossing of the South-east Indian Ridge (150-160°E), downstream of the Pacific Fracture Zone (90-140°W) (Supplementary Information Fig. 1). Note that during interaction with topography, steering also leads to meandering of the jets, especially around the Campbell Plateau (170-180°E). However, SRWs are not well represented in the 1° resolution model.

Theory

Following a simple one layer quasi-geostrophic model, the dispersion relation of a Rossby wave in a flow is [38]:

$$\omega = \bar{u}k - \frac{\beta k}{k^2 + l^2}$$

$$\beta = 2\,\Omega\, cos(\theta)/a$$

With $\bar{u}$ the flow velocity, Ω the angular speed of the Earth's rotation, a the mean radius of the Earth and θ the latitude and (k, l) the wave number.

It is possible to have stationary waves ($\omega = 0$) for a westerly flow:

$$\bar{u} = L_S\beta/4\pi^2$$

With L_S the wavelength of the wave.

To obtain wavelength between 510km (between 30-60°E) and 320km (between 90-140°W), the linear theory tells us that the westerly flow should be between 11.6 cm/s and of 3.4 cm/s. The average velocity between 1000m and the bottom are close to these theoretical values (not shown).

Supplementary Methods 3: **Ocean and BGC models' parameterizations and spin up procedure**

In the 1/10° simulation, horizontal mixing is provided by the biharmonic Smagorinsky viscosity scheme [61], and vertical mixing by the K-profile parameterization (KPP [62]). Below 2000m, a non-adaptive relaxation keeps the deep-ocean close to the observed climatology but allows climate change signals to penetrate to the deep ocean. The 1° ocean model uses the KPP scheme and a modified skew diffusive flux form [63] of the Gent and McWilliams neutral sub-grid scale eddy advection parameterisation [64].

The BGC parameters used with WOMBAT are based on [65], with extra parameters for the carbon cycle [66]. The model BGC fields are initialised with fields constructed from observations. Specifically, the World Ocean Atlas is used to initialise nutrients (phosphorus) and oxygen [67,68]. The Global Ocean Data Analysis Project (GLODAPv1) is used to initialise alkalinity and dissolved carbon [12,36]. Phytoplankton is initialised with SeaWIFS observations (NASA, 2014) and zooplankton is initialised as a fraction of phytoplankton (0.05).

For both simulations, the air-sea fluxes are calculated using bulk formula [69] for wind stress, turbulent sensible and latent fluxes, and evaporation. As the models are not coupled to a sea-ice model, the effects of sea-ice on heat and freshwater fluxes are accounted for by the use of the JRA-55 sea-ice coverage field to mask the applied atmospheric fields.

Both 1/10° and 1° simulations are initialised after a 20-year spin-up using a repeat 1979-year. That way we insure that subduction mechanism in both simulations act on a quasi-similar T-S distribution at the base of the mixed layer. As C_{ant} is calculated as the difference between two tracers of Dissolved Inorganic Carbon, C_{ant} is effectively a de-drifted passive tracer. We acknowledge that the spin-up is too short for the physics and that slow density drift occur in these simulations.

Supplementary Notes 1**: Model Validation**

The high resolution model circulation (Supplementary Information Fig. 1), climatological winter mixed layer (Supplementary Information Fig. 2) and C_{ant} distribution (Supplementary Information Fig. 3) are in good agreement with observations. The 1993-2014 time-mean geostrophic velocities and SSH products were produced by Ssalto/*Duacs* and distributed by *Aviso*, with support from *CNES* (http://www.aviso.altimetry.fr/duacs/). In the Aviso products and the high-resolution model outputs, standing meanders are located downstream of topographic obstacles, but are poorly represented in low-resolution model outputs. Vertical velocities of 65 m.day^{-1} are associated with the meanders in the high-resolution model which is in agreement with observational estimates of 70 m.day^{-1} near the Kerguelen Plateau [29].

The Argo-only 2009 CARS climatology [34,35] is distributed by CSIRO (http://www.marine.csiro.au/~dunn/cars2009/). Here, we updated the climatology using 2006-2015 Argo-only dataset. The climatology provides the mixed layer depth as a product. However, as the locally weighted scatter plot smoothing regression model (Lowess) tends to smooth out regional details (Supplementary Information Fig. 2a), we also calculated the winter mixed layer using the temperature and salinity fields provided by CARS (Supplementary Information Fig. 2b) which is in better agreement with the modelled mixed layer. Although the simulation has a deeper winter mixed-layer depth than calculated from the Argo-only CARS climatology, spatial patterns are well represented with deep mixed-layers found north of the Subantarctic Front in the south-east Indian-Ocean and in the central-east Pacific.

Observed distribution of C_{ant} are provided by GLODAPv1 [36] (http://cdiac.ornl.gov/oceans/glodap/GlopDV.html). In both low- and high-resolution models and observations, C_{ant} enters the interior at different density classes along the circumpolar path, with lighter classes in the Indian Ocean and denser ones in the Pacific (Supplementary Information Fig. 3). While it is difficult to distinguish between the spatial distributions of injected C_{ant} in high and low resolutions, the density distributions of the C_{ant} inventory are quite different (see Fig. 5). Due to the late initialisation of the biogeochemical models (1992, see Methods), the 1995 GLODAPv1 C_{ant} inventory is compared with the 2014 C_{ant} inventory in the models, focussing on the inventory away from the surface (below 100m) (see Fig. 5). While the total amount of C_{ant} in the Southern Ocean is quite similar in all models and observations, the high-resolution density distribution is more consistent with observations (Fig. 5). Both the observations and high-resolution simulation have a well-defined peak at 26.8 kgm^{3} which is absent from the low-resolution simulation. The low-resolution model still injects C_{ant} into the SAMW-AAIW but it is happening in a denser and much

wider density range (26.8 – 27.5 $kg.m^{-3}$). More specifically, when looking at the distribution in the different basins (Supplementary Information Fig. 4), the 1° model SAMW-AAIW are offset by approximately 0.1 kg.m-3 in the Indian sector, 0.2 in the Pacific and Atlantic sectors. Moreover, both models appear to store more C_{ant} than the observations at Drake Passage. As a consequence, SAMW-AAIW sit deeper and more south in the Pacific sector and are much more isolated from the C_{ant}-rich Subtropical Mode Waters, which are lighter than 26.5 $kg.m^{-3}$ in all the models experiments and observations (Supplementary Information Fig. 5).

Supplementary Notes 2: **Transient versus time-mean subduction**

The importance of eddy processes for the subduction of water masses has been suggested by numerous studies [18,70]. High-resolution simulations showed large contribution of transient eddies to mode water subduction in the northern hemisphere [49,71–73]. In the Southern Ocean, the time-mean lateral induction dominates the subduction of intermediate and mode water with only minor transient eddy contributions [39,40].

To calculate the transient eddy contribution, physical and C_{ant} subduction has been evaluated per density classes using daily outputs over 20 years (1994-2014).

The total physical subduction is expressed as

$$S_{tot}(\rho) = \overline{\int_{\rho_1<\rho<\rho_2} wdS + \int_{\rho_1<\rho<\rho_2} U.\nabla HdS}$$

Where the overbar denotes a time-average over 30 years of daily outputs.

The transient eddy subduction S^* is then defined as the difference between the total subduction and the subduction computed from the 30-year time-mean velocity and density fields:

$$\overline{S(\rho)} = \int_{\rho_1<\bar{\rho}<\rho_2} \bar{w}dS + \int_{\rho_1<\bar{\rho}<\rho_2} \bar{U}.\nabla HdS$$

$$S^*(\rho) = S_{tot}(\rho) - \overline{S(\rho)}$$

Similarly, the total C_{ant} subduction is:

$$S_{Cant\ tot}(\rho) = \overline{\int_{\rho_1<\rho<\rho_2} C_{ant}wdS + \int_{\rho_1<\rho<\rho_2} C_{ant}U.\nabla HdS}$$

$$\overline{S_{Cant}(\rho)} = \int_{\rho_1<\bar{\rho}<\rho_2} \overline{C_{ant}}\bar{w}dS + \int_{\rho_1<\bar{\rho}<\rho_2} \overline{C_{ant}}\bar{U}.\nabla HdS$$

$$S_{Cant}{}^*(\rho) = S_{Cant\ tot}(\rho) - \overline{S_{Cant}(\rho)}$$

The interaction of the SRWs and the bottom of the mixed layer occur at density class ranging from 26.8 to 27.3 kgm^{-3}. For this density range, we find a total physical subduction of 8.1Sv with only 5% contribution from the transient eddy subduction. And the transient eddy C_{ant} subduction account for 13% of the total C_{ant} subduction. For a larger range of density classes, from 26 to 27.4 kgm^{-3}, we find a total subduction of 24.8Sv, which is in agreement with previous studies [74]. For this larger range, the transient eddies contribute to 40% of the total subduction.

In the Southern Ocean, the background sloping isotherms allow for the outcrop of mode water density classes (26.8 to 27.1 kgm^{-3}). The standing eddies or standing meanders are then sufficient to connect the mode water density classes with the surface (in winter when thermocline is eroded). As the subtropical mode water interact with the bottom of mixed layer further north than the SRWs conduit, transient eddies play a more important role.

References

58. McDougall, T. J. & Klocker, A. An approximate geostrophic streamfunction for use in density surfaces. *Ocean Model.* **32,** 105–117 (2010).

59. Qiu, B. & Huang, R. X. Ventilation of the North Atlantic and North Pacific: Subduction Versus Obduction. *J. Phys. Oceanogr.* **25,** 2374–2390 (1995).

60. Sallée, J.-B., Speer, K., Rintoul, S. & Wijffels, S. Southern Ocean Thermocline Ventilation. *J. Phys. Oceanogr.* **40,** 509–529 (2010).

61. Griffies, S. M. & Hallberg, R. W. Biharmonic Friction with a Smagorinsky-Like Viscosity for Use in Large-Scale Eddy-Permitting Ocean Models. *Mon. Weather Rev.* **128,** 2935–2946 (2000).

62. Large, W. G., McWilliams, J. C. & Doney, S. C. Oceanic vertical mixing: A review and a model with a nonlocal boundary layer parameterization. *Rev. Geophys.* **32,** 363–403 (1994).

63. Griffies, S. M. The Gent–McWilliams Skew Flux. *J. Phys. Oceanogr.* **28,** 831–841 (1998).

64. Gent, P. R., Willebrand, J., McDougall, T. J. & McWilliams, J. C. Parameterizing Eddy-Induced Tracer Transports in Ocean Circulation Models. *J. Phys. Oceanogr.* **25,** 463–474 (1995).

65. Oschlies, A. & Schartau, M. Basin-scale performance of a locally optimized marine ecosystem model. *J. Mar.* **63,** 335–358 (2005).

66. Law, R. M. *et al.* The carbon cycle in the Australian Community Climate and Earth System Simulator (ACCESS-ESM1) – Part 1: Model description and pre-industrial simulation. *Geosci. Model Dev. Discuss.* **2015,** 8063–8116 (2015).

67. Garcia, H., Locarnini, R. & Boyer, T. in *NOAA Atlas NESDIS 63* (ed. Levitus, S.) 342 (U.S. Government Printing Office, Washington, D.C., 2006).

68. Garcia, H., Locarnini, R., Boyer, T. & Antonov, J. in *NOAA Atlas NESDIS 63* (ed. Levitus, S.) 396 (U.S. Government Printing Office, Washington, D.C., 2006).

69. Large, W. G. & Yeager, S. G. Diurnal to decadal global forcing for ocean and sea--ice models: The data sets and flux climatologies. *NCAR Tech. Note* **TN--460+ST,** 105pp (2004).

70. Marshall, D. P., Williams, R. G. & Lee, M.-M. The Relation between Eddy-Induced Transport and Isopycnic Gradients of Potential Vorticity. *J. Phys. Oceanogr.* **29,** 1571–1578 (1999).

71. Qu, T., Xie, S.-P., Mitsudera, H. & Ishida, A. Subduction of the North Pacific Mode Waters in a Global High-Resolution GCM. *J. Phys. Oceanogr.* **32,** 746–763 (2002).

72. Nishikawa, S., Tsujino, H., Sakamoto, K. & Nakano, H. Effects of Mesoscale Eddies on Subduction and Distribution of Subtropical Mode Water in an Eddy-Resolving OGCM of the Western North Pacific. *J. Phys. Oceanogr.* **40,** 1748–1765 (2010).

73. Da Costa, M. V., Mercier, H. & Treguier, A. M. Effects of the Mixed Layer Time Variability on Kinematic Subduction Rate Diagnostics. *J. Phys. Oceanogr.* **35,** 427–443 (2005).

74. Sloyan, B. M. & Rintoul, S. R. Circulation, Renewal, and Modification of Antarctic Mode and Intermediate Water. *J. Phys. Oceanogr.* **31,** 1005–1030 (2001).

Supplementary Figures

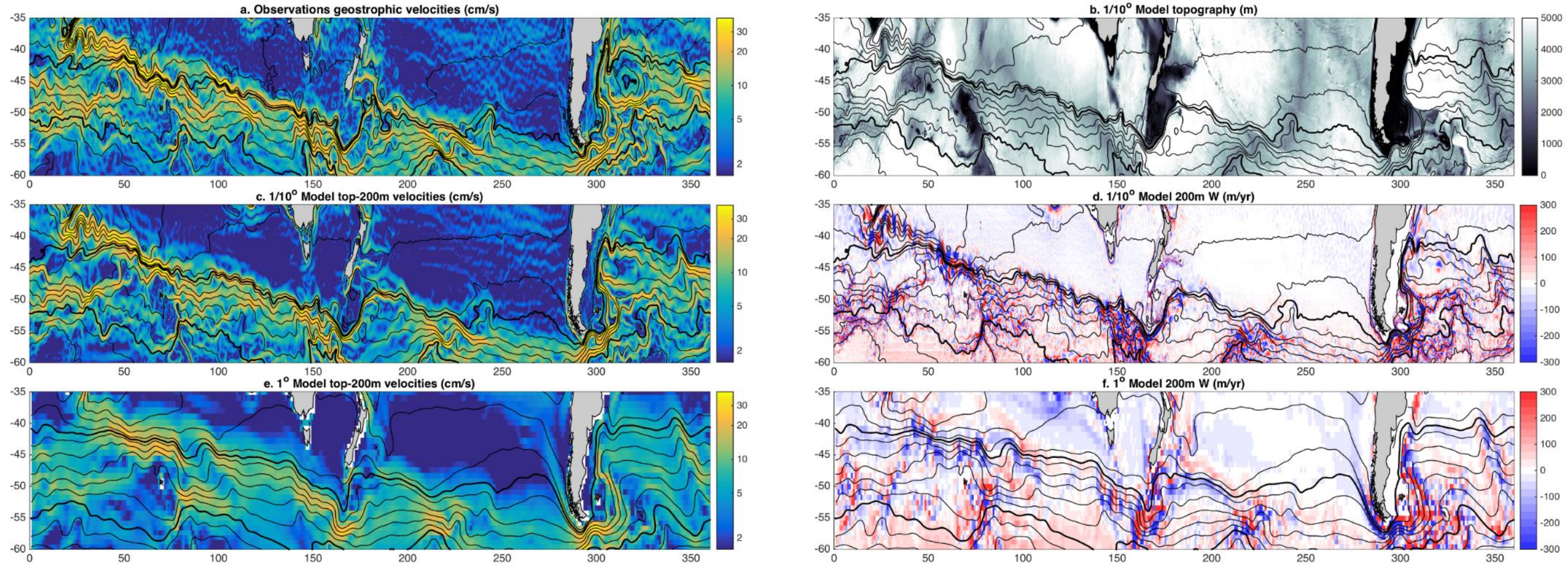

Fig. 1: **Circulation and standing meanders: impact of resolution.** a) AVISO 1993-2014 time-mean geostrophic velocities and SSH contours. b) 1/10° model topography and SSH contours. Time-mean model horizontal velocities average over the top 200m and SSH contours (left), and time-mean model vertical velocities at 200m and SSH contours (right), for 2 different resolution: 1/10° c) and d) and 1° e) and f). SAF and PF are underlined in bold. Figures are plotted using MATLAB R2015a (http://www.mathworks.com/).

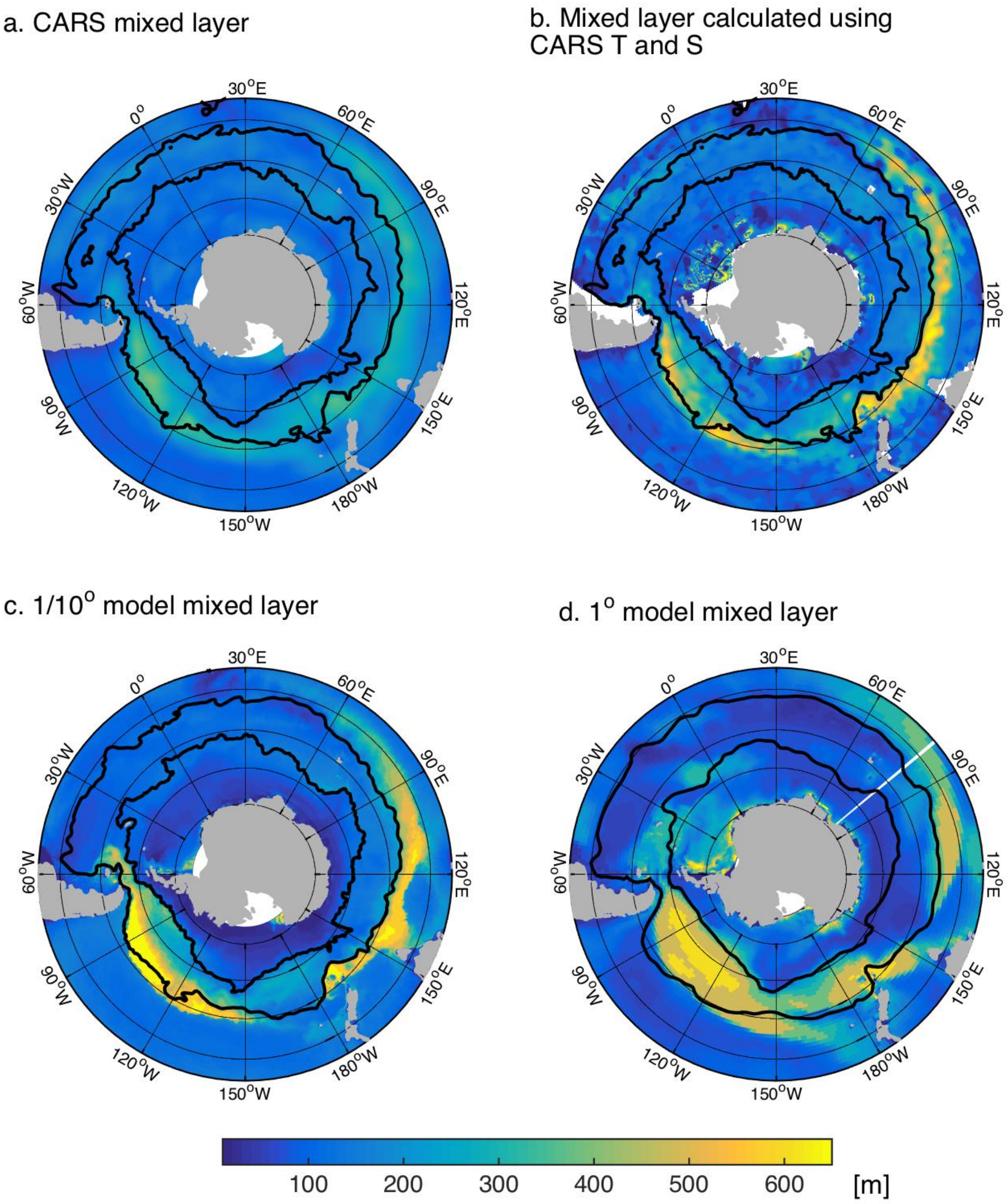

Fig. 2: **Validation of maximum winter mixed layer**: a) maximum winter mixed layer from CARS climatology (2006-2015), b) maximum winter mixed layer calculated using temperature and salinity from CARS climatology (2006-2015), c) 1/10° model climatological winter mixed layer (1981-2012) and d) 1° model climatological winter mixed layer (1981-2012). The mixed layer is calculated using surface density difference criterion of 0.03 kg m^{-3}. SSH contours show the position of SAF and PF. . Figures are plotted using MATLAB R2015a (http://www.mathworks.com/). The maps are generated using M_Map (a mapping package, http://www.eos.ubc.ca/~rich/map.html).

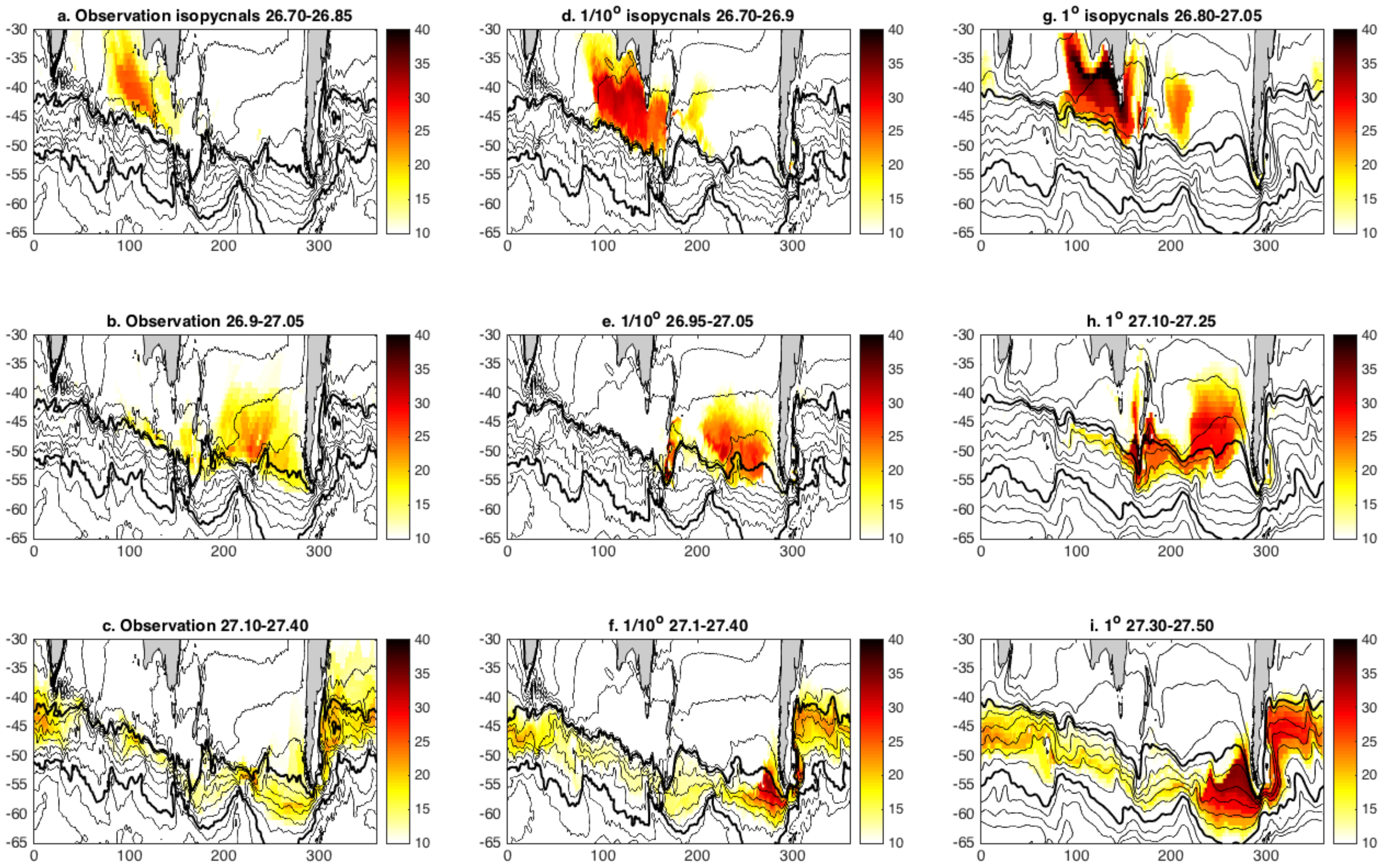

Fig. 3: **Validation of Carbon inventory in the Southern Ocean:** C_{ant} inventory of GLODAPv1 (a b and c), 1/10° model (d e and f)) and 1° model (g h and i) along selected isopycnal surfaces. Black lines are SSH contours with SAF and PF in bold. The isopycnal surfaces has been chosen to fit the inventory peaks in each basin (see Extended data Fig.4). Note that the isopycnals surface are denser for 1° model. Due to how the biogeochemical models were initialised (see Methods), the 1995 observations are compared with the 2014 C_{ant} inventory in the models. Figures are plotted using MATLAB R2015a (http://www.mathworks.com/).

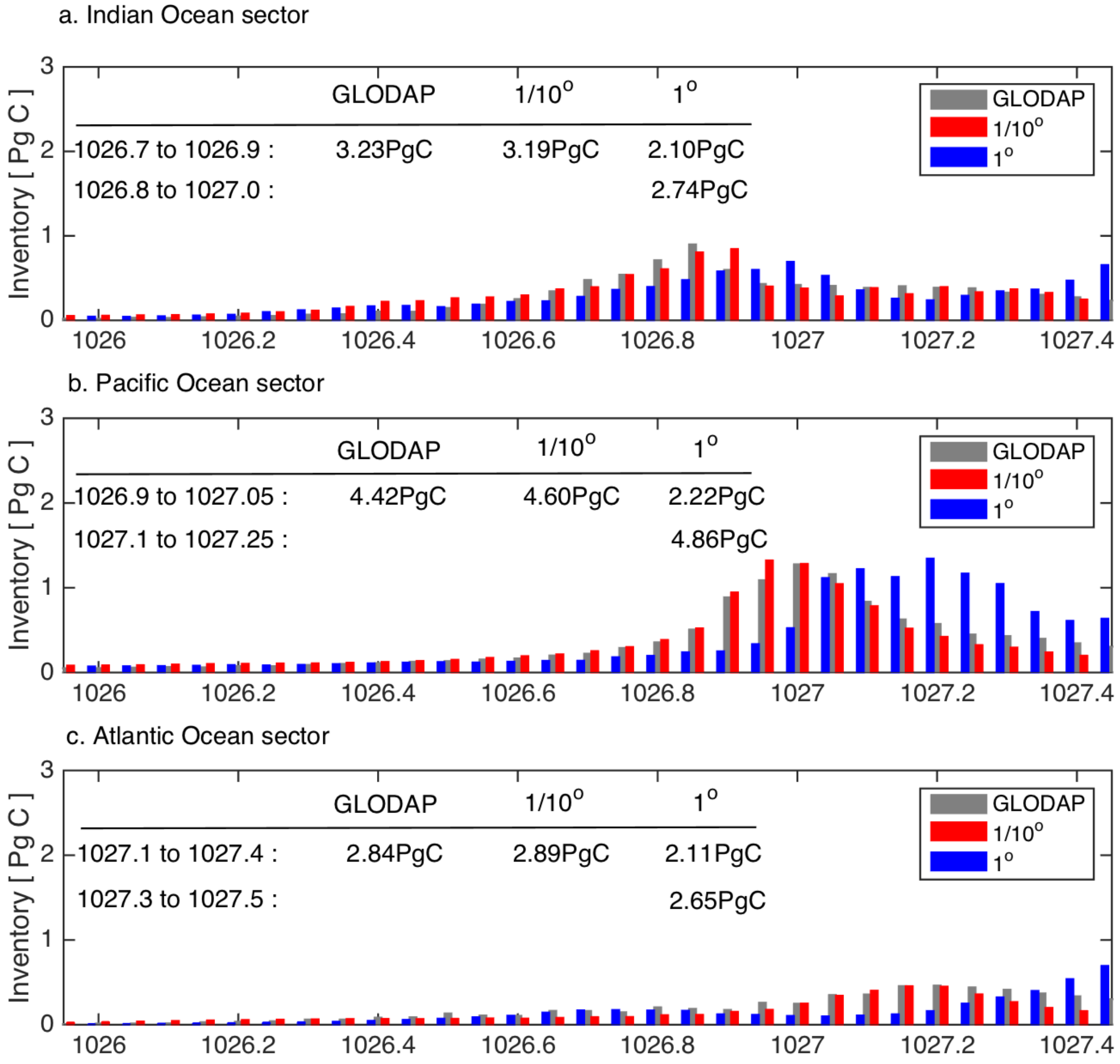

Fig. 4: **C_{ant} inventory in different ocean basin**: below 100m and between 30°S and the PF for comparison between GLODAP 1995 (grey bars), 1/10° model (red bars) and 1° model (blue bars) for 2014. Note the inventory in denser layers in the 1° model and how the density classes of the inventory maximum shift towards denser values from the Indian basin to the Pacific and Atlantic basins. Figures are plotted using MATLAB R2015a (http://www.mathworks.com/).

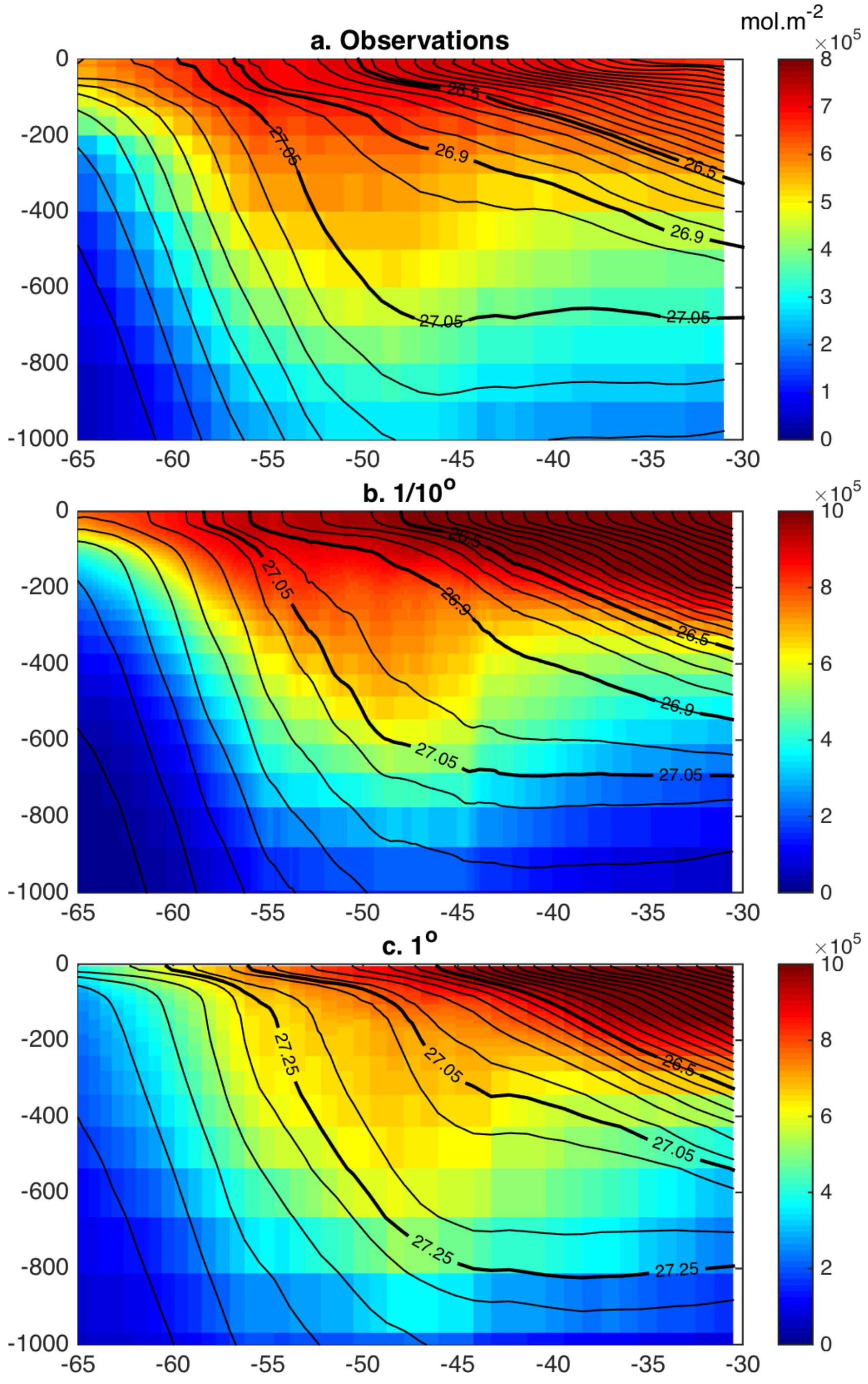

Fig. 5: **Connection between SAMW-AAIW and Subtropical Mode Waters in the pacific**: Zonal-mean (145-300°E) C_{ant} inventory in the Pacific sector (colour) along with isopycnals contours for GLODAP observations a), 1/10° model b), and 1° model c). From the surface to the deep ocean, bold contours define the lower limit of C_{ant}-rich Subtropical Mode Water and the limits of the SAMW-AAIW. Note that, while the Subtropical Mode waters limit is similar for the 3 products (isopycnals 26.5kg.m^{-3}) denser isopycnals delimit the SAMW-AAIW for the 1° model. As the modelled C_{ant} inventories are presented for 2014 (see models initialisation in Methods), modelled Subtropical Mode Waters have a higher C_{ant} storage in comparison with observations. Figures are plotted using MATLAB R2015a (http://www.mathworks.com/).

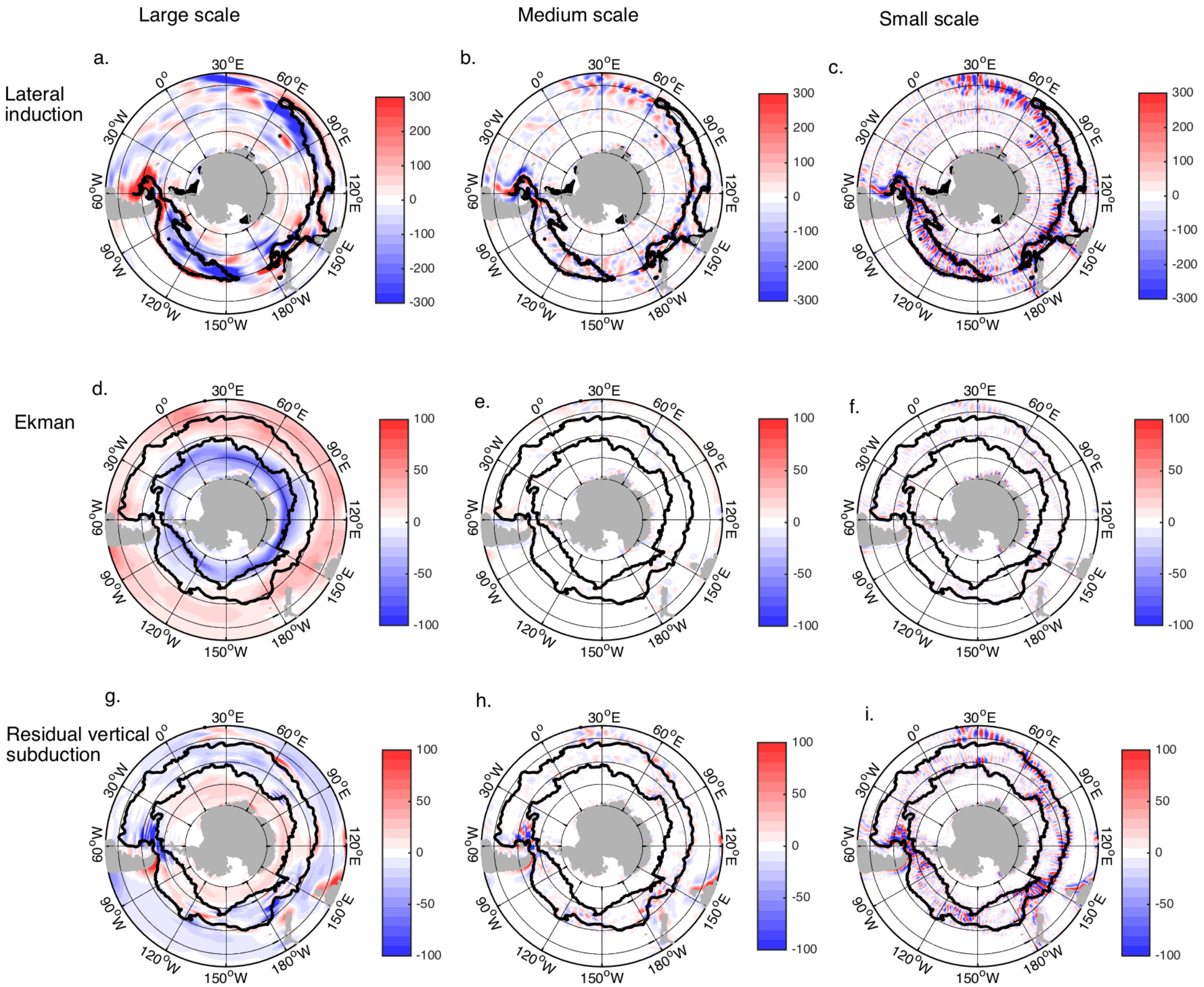

Fig. 6: **Scales separations of the Physical transfers** into (+) and out (-) the ocean interior at the base of the winter mixed layer in 1/10° model for lateral induction (a, b and c), Ekman pumping (d, e and f) and residual vertical eddy-induced subduction (g, h and i). Black lines in d) to i) show the mean position of the SAF and PF. Black contours in a), b) and c) show the position of 300m winter mixed layer. Figures are plotted using MATLAB R2015a (http://www.mathworks.com/). The maps are generated using M_Map (a mapping package, http://www.eos.ubc.ca/~rich/map.html).

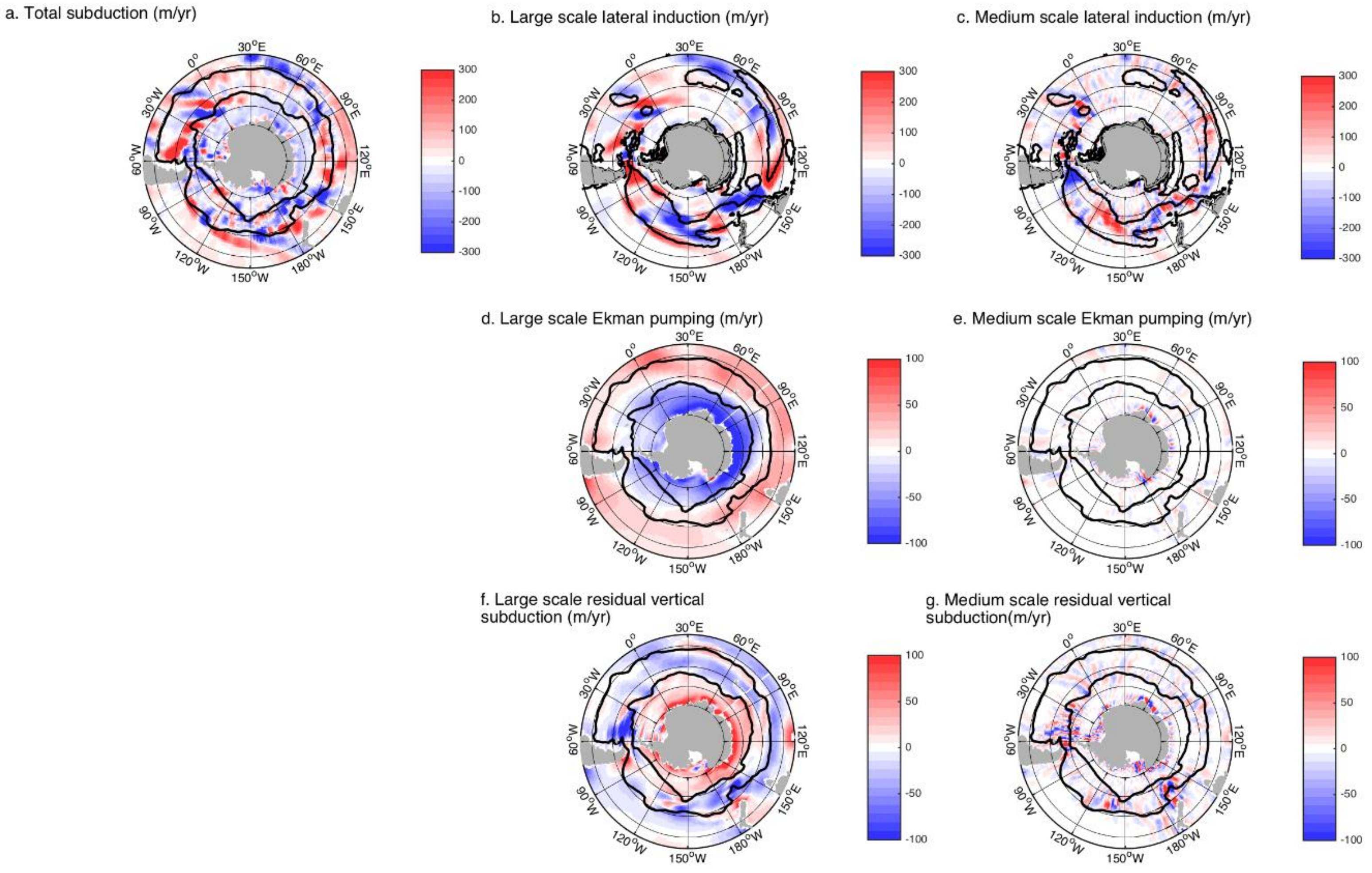

Fig. 7: **Scales separations of the Physical transfers** into (+) and out (-) the ocean interior at the base of the winter mixed layer in 1° model for lateral induction (b and c), Ekman pumping (d and e) and residual vertical eddy-induced subduction (f and g). Black lines in d) to g) show the mean position of the SAF and PF. Black contours in a), b) and c) show the position of 300m winter mixed layer. Figures are plotted using MATLAB R2015a (http://www.mathworks.com/). The maps are generated using M_Map (a mapping package, http://www.eos.ubc.ca/~rich/map.html).

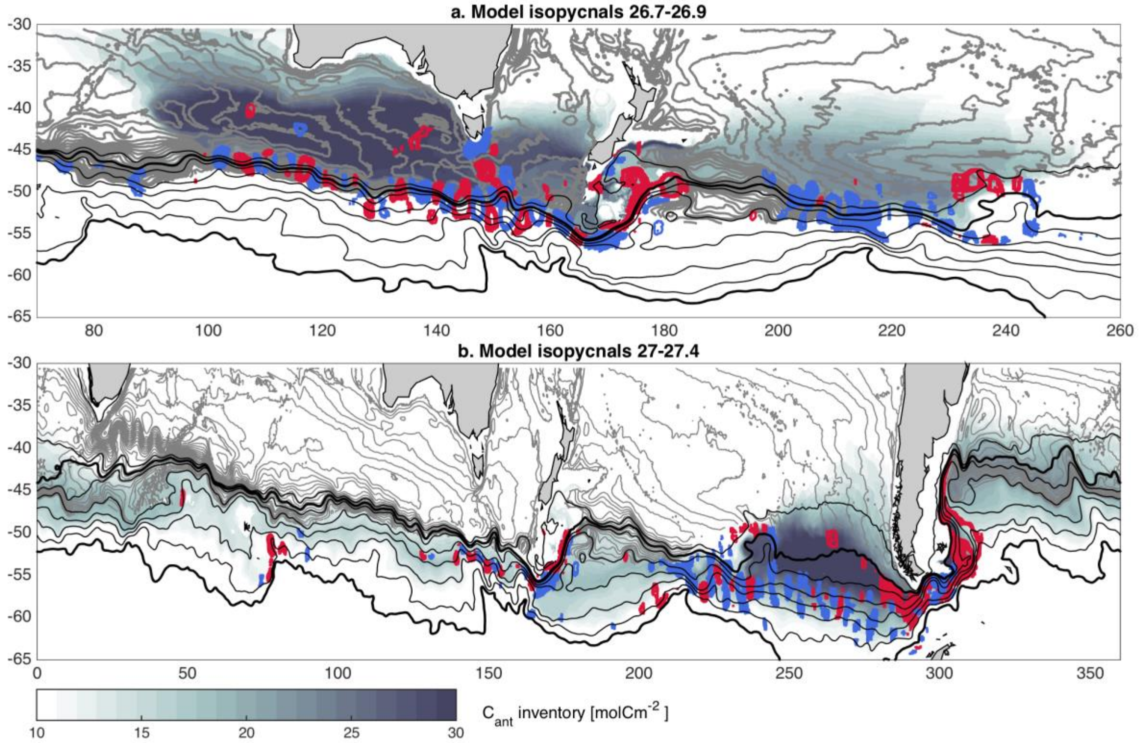

Fig. 8: **Colocation of stationary Rossby waves induced subduction and C_{ant} inventory along selected isopycnal surfaces**: 26.7-26.9 a) and 27-27.4 kg m^{-3} b). The grey shades show the inventory and the grey contours show the approximate geostrophic stream-function along the selected isopycnals as defined by [58]. Black lines are SSH contours with SAF and PF in bold. Blue and red contours are lateral induction transport (as in Fig 1b), shown only where selected isopycnals outcrop at the bottom of the winter mixed-layer. Figures are plotted using MATLAB R2015a (http://www.mathworks.com/).

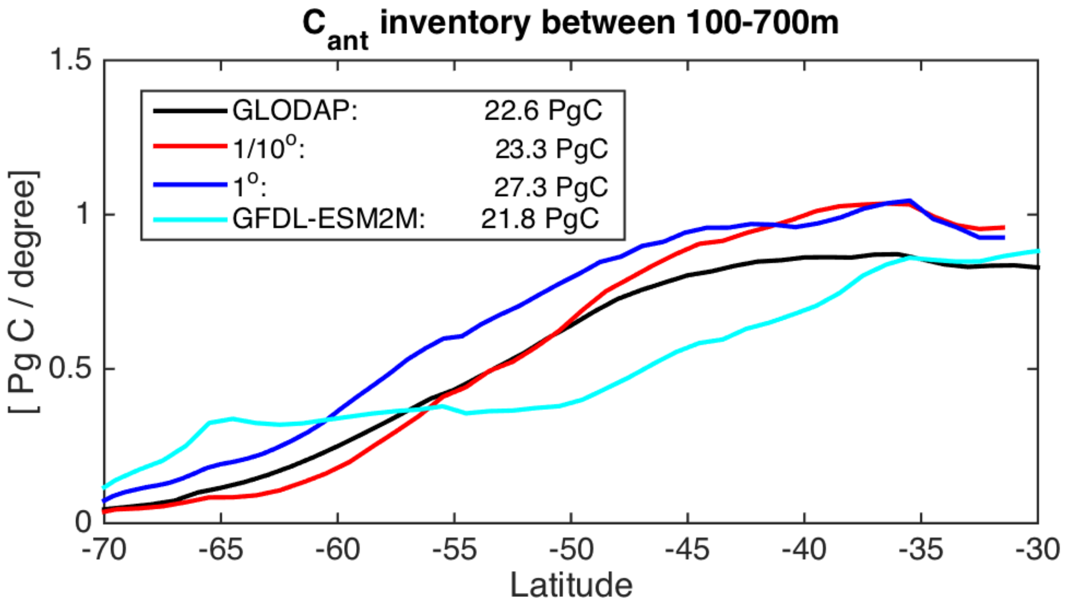

Fig. 9: **Anthropogenic carbon storage**: zonal integrated oceanic anthropogenic carbon storage in the intermediate ventilated layer 100-700m. Due to the late initialisation of the biogeochemical models (1993, see Methods), 1995 observations are compared with the 2014 C_{ant} inventory in the models. For GFDL-ESM2m, the storage is calculated as in Frolicher et al., 2015 as a change of carbon between 1870 and 1995. Figures are plotted using MATLAB R2015a (http://www.mathworks.com/).

Supplementary table

Resolution	Scales	26.8 < σ < 27.3	26.5 < σ < 28
1°	All	0.46 $PgCyr^{-1}$	0.80 $PgCyr^{-1}$
	Large	0.29 $PgCyr^{-1}$	0.35 $PgCyr^{-1}$
	Medium	0.17 $PgCyr^{-1}$	0.45 $PgCyr^{-1}$
1/10°	All	1.02 $PgCyr^{-1}$	1.45 $PgCyr^{-1}$
	Large	0.18 $PgCyr^{-1}$	0.18 $PgCyr^{-1}$
	Medium	0.10 $PgCyr^{-1}$	0.15 $PgCyr^{-1}$
	Small	0.74 $PgCyr^{-1}$	1.12 $PgCyr^{-1}$

Table 1**: C_{ant} subduction rate through the base of the winter mixed layer** in Pg C yr^{-1} south of 30°S for different range of density classes.
